# Identification and analysis of major latex protein (*MLP*) family genes in *Rosa chinensis* responsive to *Botrytis cinerea* infection by RNA-seq approaches

**DOI:** 10.3389/fpls.2024.1511597

**Published:** 2024-12-13

**Authors:** Haoyuan Chen, Qingkui Li, Peilei Cheng, Taotao Yan, Chunlan Dong, Zhe Hou, Peihuang Zhu, Changbing Huang

**Affiliations:** ^1^ College of Horticultural Science and Technology, Suzhou Polytechnic Institute of Agriculture, Suzhou, China; ^2^ College of Landscape Engineering, Suzhou Polytechnic Institute of Agriculture, Suzhou, China

**Keywords:** major latex protein, rose, RNA-Seq, tandem duplication, *Botrytis cinerea*

## Abstract

Roses (*Rosa chinensis*) are among the most cherished ornamental plants globally, yet they are highly susceptible to infections by *Botrytis cinerea*, the causative agent of gray mold disease. Here we inoculated the resistant rose variety ‘Yellow Leisure Liness’ with *B. cinerea* to investigate its resistance mechanisms against gray mold disease. Through transcriptome sequencing, we identified 578 differentially expressed genes (DEGs) that were significantly upregulated at 24, 48, and 72 hours post-inoculation, with these genes significantly enriched for three defense response-related GO terms. Further domain analysis of the genes in these GO terms reveal that 21 DEGs contain the Bet v 1 family domain, belonging to the major latex protein (*MLP*) gene family, suggesting their potential key role in rose disease resistance. Furthermore, we systematically identified 46 *RcMLP* genes in roses and phylogenetically categorized them into two distinct subfamilies: group I and II. Genomic duplication analysis indicates that tandem duplication is the main driver for the expansion of the *RcMLP* family, and these genes have undergone by purifying selection. Additionally, detailed analyses of gene structure, motif composition, and promoter regions reveal that *RcMLP* genes contain numerous stress-responsive elements, with 32 *RcMLP* genes harboring fungal elicitor/wound-responsive elements. The constructed potential transcription factor regulatory network showed significant enrichment of the ERF transcription factor family in the regulation of *RcMLP* genes. Gene expression analysis reveal that DEGs are mainly distributed in subfamily II, where four highly expressed genes (*RcMLP13*, *RcMLP28*, *RcMLP14*, and *RcMLP27*) are identified in a small branch, with their fold change exceeding ten folds and verified by qRT-PCR. In summary, our research results underscore the potential importance of the *RcMLP* gene family in response to *B. cinerea* infection and provide comprehensive basis for further function exploration of the *MLP* gene family in rose resistance to fungal infections.

## 1 Introduction

*Rosa chinensis*, commonly known as the Chinese rose, is highly valued as both a garden ornamental and a source for cut flowers. It holds a pivotal role in horticulture and the economy, especially being indispensable in perfumery and cosmetic industries (Bendahmane et al., 2013; Mileva et al., 2021; Shang et al., 2024). However, the splendor and economic worth of roses are frequently threatened by various pathogens, with *Botrytis cinerea* being a particularly catastrophic fungus, notorious for causing gray mold disease across over 1000 plant species (Dean et al., 2012). This necrotrophic fungus can rapidly lead to significant crop losses, affecting not only the aesthetic appeal of roses but also their marketability (Ullah et al., 2024).

Given the enduring significance of roses in the floriculture industry, there is a pressing need to unravel the molecular mechanisms underlying their defense against such pathogens. Recent research has gradually uncovered how roses combat diseases through complex molecular mechanisms. For instance, the WRKY gene family has demonstrated potential in enhancing the disease resistance of roses (Liu et al., 2019, Liu et al., 2023). Major latex-like proteins (MLPs) homologs can be divided into three groups: MLPs, Bet v 1s, and pathogenesis-related proteins class 10 (PR-10s), one of the 17 members of the PR family (Fujita and Inui, 2021). These regulatory proteins are responsive to biotic and abiotic stress and involved in various physiological and biochemical processes, including responses to drought, salt, plant hormones, and pathogen infections (Fujita and Inui, 2021). They have been identified in many plants, such as *Malus domestica* (Yuan et al., 2020), *Cucumis sativus* (Kang et al., 2023), peanut (Li et al., 2023), Populus (Sun et al., 2024). A recent study identified the *MLP* gene family in *Pyrus bretschneideri*, with *PbrMLP* genes deemed as vital candidates for resistance to *Colletotrichum fructicola* in pears (Su et al., 2024a). Furthermore, a handful of studies have established the PR-10 proteins’ role as a signaling module in defense against *Botrytis cinerea*, outlining regulatory mechanisms such as the ‘Ethylene-MPK8-ERF.C1-PR’ module and the RhERF005/RhCCCH12–RhPR10.1 module, which mediate resistance and cytokinin-induced defense responses, respectively (Deng et al., 2024; Liu et al., 2024). Proteomic analysis has uncovered the role of four PR10 proteins and a plasma membrane aquaporin in the rose defense against *Botrytis cinerea* infection (Li et al., 2024). These studies highlight the potential of *MLP* gene family as significant regulators in response to fungal infections. However, a comprehensive identification of *MLP* genes in roses and an in-depth exploration of their expression patterns in response to *Botrytis cinerea* infection remain underexplored.

In this study, leveraging publicly available genomic data, we analyzed the transcriptional changes in rose petals during *B. cinerea* infection, identifying 578 significantly upregulated DEGs at 24, 48, and 72 hours post-infection. Gene Ontology (GO) analysis of these DEGs reveals a significant enrichment of defense response-related terms among *RcMLP* genes. Furthermore, we systematically identified and analyzed 46 *RcMLP* genes, including their gene structure, motif composition, evolutionary relationships, chromosomal localization, collinearity analysis, and transcription factor regulatory networks, highlighting the potential significance of the *MLP* gene family in rose resistance to fungal infection. Our research not only provides new insights into the genomic evolutionary characteristics of the *MLP* gene family but also lays an essential foundation for exploring their expression patterns and functional mechanisms under pathogen invasion.

## 2 Materials and methods

### 2.1 Plant materials and fungal growth and plant infection

Rose cultivars were grown in greenhouses in Suzhou Polytechnic Institute of Agriculture (Jiangsu, China), under the controlled conditions of 75% relative humidity and 25°C under a 16 h light and 8 h dark photoperiod. An *B. cinerea* strain was isolated from typical diseased petals and then stored in 15% (v/v) glycerol at −80°C. *B. cinerea* was cultured on potato glucose agar (PDA) media in 25°C growth chamber for about 2 weeks. The inoculum was prepared by harvesting *B. cinerea* spores with ddH_2_O and then suspending in 1/2 PDB to a final concentration of 1×10^5^ conidia/mL. For inoculation, *B. cinerea* inoculum were sprayed evenly on the flowers, with 1 mL inoculum per flower, then the inoculated flowers were covered with plastic bags to ensuren100% humidity. A comprehensive screening for resistance to *B. cinerea* was conducted across all cultivars with stable phenotypic traits in the germplasm repository at Suzhou Agricultural Vocational College under uniform irrigation and fertilization practices. This systematic evaluation led to the identification of two distinct rose varieties with markedly different responses to the disease: “Yellow Leisure Liness” which demonstrated high resistance, and “Miyaho,” which exhibited sensitivity to gray mold. Control and infected petals for two cultivars were individually sampled in a randomized manner at 0 h, 24 h, 48 h and 72 h, with three biological repeats at each time point. Petals were immediately frozen in liquid nitrogen at the time of harvesting and stored at −80°C.

### 2.2 RNA-seq library construction

Following the OMEGA RNA kit protocol, total RNA was extracted from rose petals with three replicates. Subsequently, the purity, concentration and integrity of RNA samples were examined by NanoDrop, Qubit 2.0, Agilent 2100, *etc.*, respectively. Qualified RNA was processed for library construction. Subsequently, the first-strand of cDNA was synthesized with fragmented mRNA as template and random hexamers as primers, followed by second-strand synthesis with addition of PCR buffer, dNTPs, RNase H and DNA polymerase I. Purification of cDNA was processed with AMPure XP beads. Then, Double-strand cDNA was subjected to end repair. Adenosine was added to the end and ligated to adapters. AMPure XP beads were applied here to select fragments within size range of 300**–**400 bp. Finally, cDNA library was obtained by certain rounds of PCR on cDNA fragments generated from on step. In addition, to ensure the quality of library, Qubit 2.0 and Agilent 2100 were used to examine the concentration of cDNA and insert size. The libraries were then sequenced by on Illumina platform with PE150 mode.

### 2.3 Reads processing and differentially expressed genes analysis

It is crucial to ensure the quality of the reads before moving onto following analysis. Low quality sequences, primers were removed by BMKCloud (www.biocloud.net) and clean data were collected. Then, clean reads were aligned to the reference genome *R. chinensis* ‘Old Blush’ (v2.0) by HISAT2 v2.2.1 software (Kim et al., 2015). The *R. chinensis* genome was downloaded from Genome Database for Rosaceae (https://www.rosaceae.org/). The read count for each gene was determined by the StringTie v2.2.0 (Pertea et al., 2015), and the FPKM (Fragments Per Kilobase of transcript per Million fragments mapped) was used to quantification the expression level of each gene. Furthermore, DEGs were identified based on the criteria of an absolute log2Ratio > 1 and the false discovery rate (FDR) < 0.01, utilized the R package DESeq2 (Love et al., 2014). Additionally, GO enrichment and KEGG pathway analyses were performed to explore the biological functions of the DEGs, utilizing clusterProfiler (Yu et al., 2012) and KOBAS software (Xie et al., 2011), respectively.

### 2.4 Genome-wide identification and characterization of *RcMLP* genes

To identify candidate members of the *MLP* genes within the *R. chinensis* ‘Old Blush’ (v2.0) genome, the hidden Markov model (HMM) profile for the Bet_v_1 (PF000407) domain was retrieved from the Pfam database (http://pfam.xfam.org/) database. All rose genes were then screened using HMMER 3.0 (http://hmmer.org/), with an E-value threshold of < 1e−10, and those containing the Bet_v_1 domain were designated as candidate *RcMLP* genes. Further validation of the conserved domain in these candidate *RcMLP* genes was conducted using the SMART database (https://smart.embl.de/) and the NCBI-CDD platform (https://www.ncbi.nlm.nih.gov/Structure/cdd/cdd.shtml) to ensure accuracy. Characteristics of the *RcMLP* genes, including amino acid length, molecular weight (MW), theoretical isoelectric point (PI), and grand average of hydropathicity (GRAVY), were analyzed using the ExPASy website (http://web.expasy.org/protparam/) (Artimo et al., 2012). Additionally, the predicted subcellular locations of these genes were determined using the Plant-mPLoc tool (http://www.csbio.sjtu.edu.cn/bioinf/plant-multi/).

### 2.5 Multiple sequences alignment, phylogenetic analysis, and tertiary structure prediction of *RcMLP* genes

To explore the evolutionary relationships of *MLPs*, sequences from *R. roxburghii*, *Oryza sativa*, and *Arabidopsis thaliana* were retrieved from the CNCB (https://ngdc.cncb.ac.cn/gwh/Assembly/84056/show), Phytozome (https://phytozome-next.jgi.doe.gov/), and TAIR (https://www.arabidopsis.org/) databases, respectively. Subsequently, the complete MLP protein sequences from these four species were aligned using MAFFT (Katoh and Toh, 2008) v7.4.1. An un-rooted phylogenetic tree was constructed using the maximum likelihood (ML) method in MEGA 11 (Kumar et al., 2018) with 1000 replicates boot-strap test. The tree was further refined and visualized on the Evolview v3 (Subramanian et al., 2019) platform (https://www.evolgenius.info/evolview/). Additionally, the conserved domains Bet_v_1 of all *RcMLPs* were annotated and visualized using the ggMSA software (Zhou et al., 2022) with default parameters. The tertiary structure of typical Bet_v_1 domain was retrieved from the AlphaFold Protein Structure Database (https://alphafold.ebi.ac.uk/).

### 2.6 Chromosome location and gene syntenic analysis of *RcMLP* genes

The physical locations of the *RcMLP* genes across various chromosomes were determined from the gff annotation of the rose genome and visualized using TB-tools with advanced Circos options (Chen et al., 2020). Gene duplication events among the *RcMLP* genes were identified using MCScanX (Wang et al., 2012). Furthermore, the rates of nonsynonymous substitution (Ka), synonymous substitution (Ks), and the Ka/Ks ratio for the duplicated *RcMLP* genes were calculated with KaKs_Calculator 3.0 (Wang et al., 2010). Genes with a Ka/Ks ratio greater than 1 are considered to be under positive selection; those with a Ka/Ks ratio equal to 1 are considered neutral; and those with a Ka/Ks ratio less than 1 are considered to be under negative or purifying selection.

### 2.7 Gene structure, conserved motif and *cis*-element analysis of *RcMLP* genes

The exon and intron sequences of the *RcMLP* genes were extracted from the gff annotation file of the rose genome. Following this, the web-based tool MEME v5.5.0 (http://meme-suite.org/tools/meme) was utilized to identify conserved motifs, employing all default parameters. Subsequently, PlantCARE (http://bioinformatics.psb.ugent.be/webtools/plantcare/html/) was used to scan the 2-kilobase pair upstream regions of each *RcMLP* gene for the presence of potential *cis*-acting regulatory elements.

### 2.8 Transcription factor regulatory network analysis of *RcMLP* genes

Potential regulatory interactions involving transcription factors (TFs) within the 2-kilobase pair upstream regions of candidate *RcML*P genes were predicted using the Plant Transcriptional Regulatory Map (PTRM, http://plantregmap.gao-lab.org/), with *Arabidopsis thaliana* as the reference species and a screening threshold of P ≤ 1e^−5^. Visual representations of the predicted TF networks using Cytoscape software (Shannon et al., 2003). The wordcloud is generated by the ggplot2 package. Furthermore, we also conducted protein-protein interaction (PPI) networks analysis by RcMLP protein sequences by STRING (https://cn.string-db.org/).

### 2.9 Quantitative real-time PCR analysis

To validate the results from the RNA-Seq assay, 6 DEGs with great alterations were chosen and validated by qRT-PCR. The flowers of ‘Yellow Leisure Liness’ were evenly sprayed with *B. cinerea*, and the petals were collected at 0 h, 24 h, 48 h and 72 h to detected the expression. The primers for the candidate DEGs and *GAPDH* gene were designed by Primer 5.0 software and are shown in **Supplementary Table S1**. Following the standard protocol of the ABI7500 system, the amplification programs for candidate genes in triplicate were validated by qRT-PCR, and the relative quantitative method (2^−△△CT^) was used to calculate the fold changes to define the expression levels of target genes (Schefe et al., 2006).

## 3 Results

### 3.1 Transcriptome profiling of rose in response to *B. cinerea* infection

**Figure 1:**
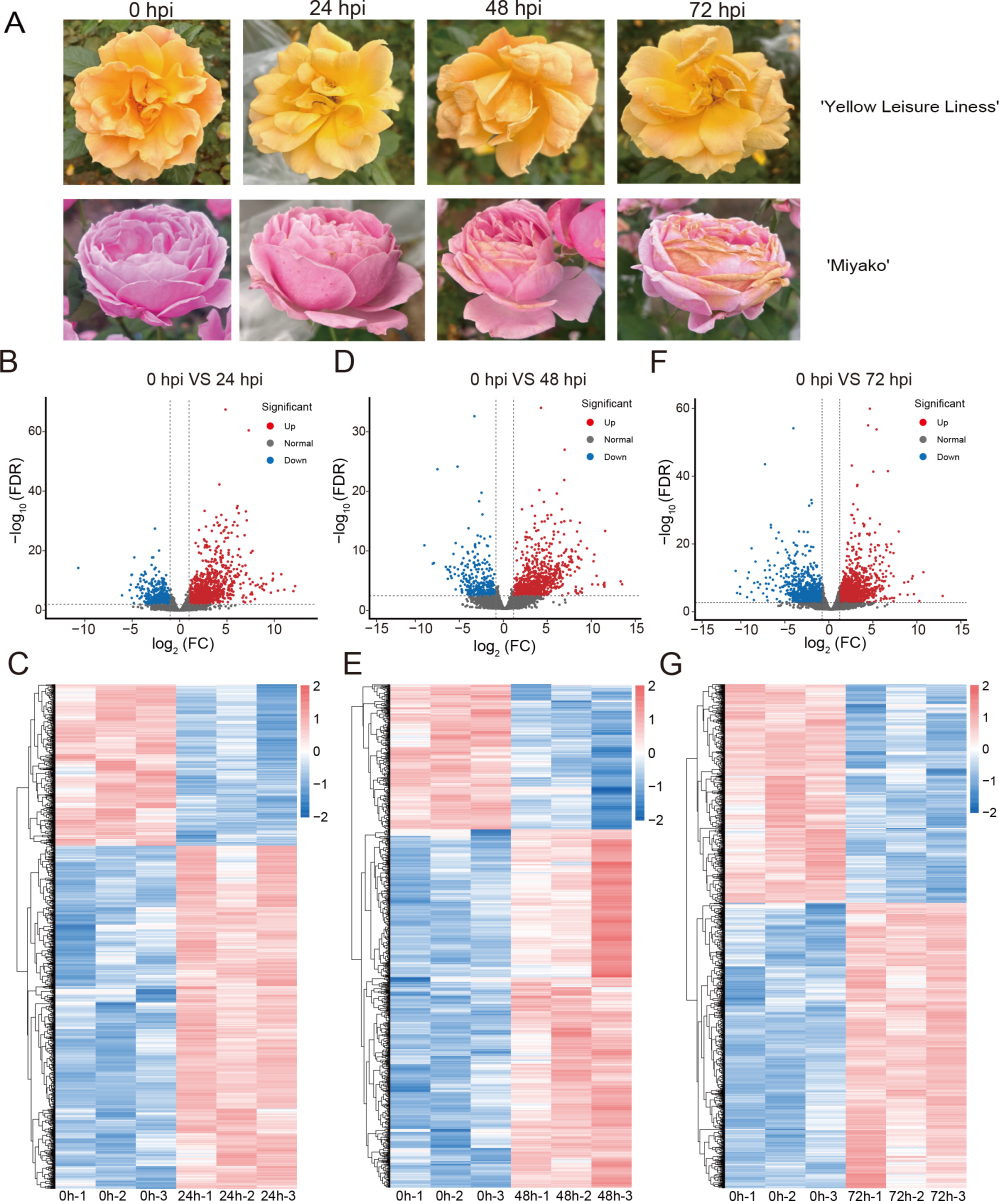
Development of *B. cinerea* on rose petal and analysis of DEGs in roses across control and time-based infections. **(A)** Variably severe disease lesions observed in two rose cultivars post-inoculation under different time of treatment. The volcano plots show DEGs of ‘Yellow Leisure Liness’ cultivar between uninfected rose petals (0 hour) and infected by *B. cinerea* at 24 hours **(B)**, 48 hours **(D)**, and 72 hours **(F)**, based on the criteria of log2 fold change (FC) > 1 and FDR value < 0.01. Heatmaps show the expression levels of DEGs in different samples at 24 hours **(C)**, 48 hours **(E)**, and 72 hours **(G)** post-infection with *B. cinerea*. Data were homogenized by Z-score.

Through inoculation with *B. cinerea*, we have identified two different resistant rose cultivars, ‘Yellow Leisure Liness’ and ‘Miyako’ (**Figure 1A**). The ‘Yellow Leisure Liness’ cultivar exhibited exceptional resistance, with no noticeable disease spots on the petals even 48 hours post-inoculation (hpi). In contrast, the ‘Miyako’ cultivar had begun to show expanding lesions after 24 hpi. The ‘Miyako’ cultivar’s spots were significantly more severe than those on ‘Yellow Leisure Liness’ at all teat time points, suggesting that ‘Yellow Leisure Liness’ has a decreased susceptibility to the pathogen, marking it as a resistant cultivar.

To elucidate the resistance mechanisms of the ‘Yellow Leisure Liness’ rose cultivar, we selected uninfected and treated at 24, 48, and 72 hpi petals for comprehensive transcriptomic sequencing analysis. The sequencing yielded a total of 52,906,936 reads, with at least 81.14% aligning to the reference genome (**Supplementary Table S2**). This dataset allowed us to identify DEGs in infected petals compared to the controls, with criteria set at a log2 fold change (FC) > 1 and FDR < 0.01.

At 24 hpi, we identified 1,797 DEGs, with 1,211 upregulated and 586 downregulated (**Figures 1B, E**; **Supplementary Table S3**). At 48 hpi, the number of significantly altered DEGs was 1,200, including 850 upregulated and 350 downregulated (**Figures 1C, F**; **Supplementary Table S4**). By 72 hpi, the number of DEGs had risen to 2,449, with 1,381 upregulated and 1,068 downregulated (**Figures 1D, G**; **Supplementary Table S5**). These DEGs are considered part of the rose’s response repertoire to *Botrytis cinerea* infection.

### 3.2 *MLP* genes are involved in rose resistance to *B. cinerea*

**Figure 2:**
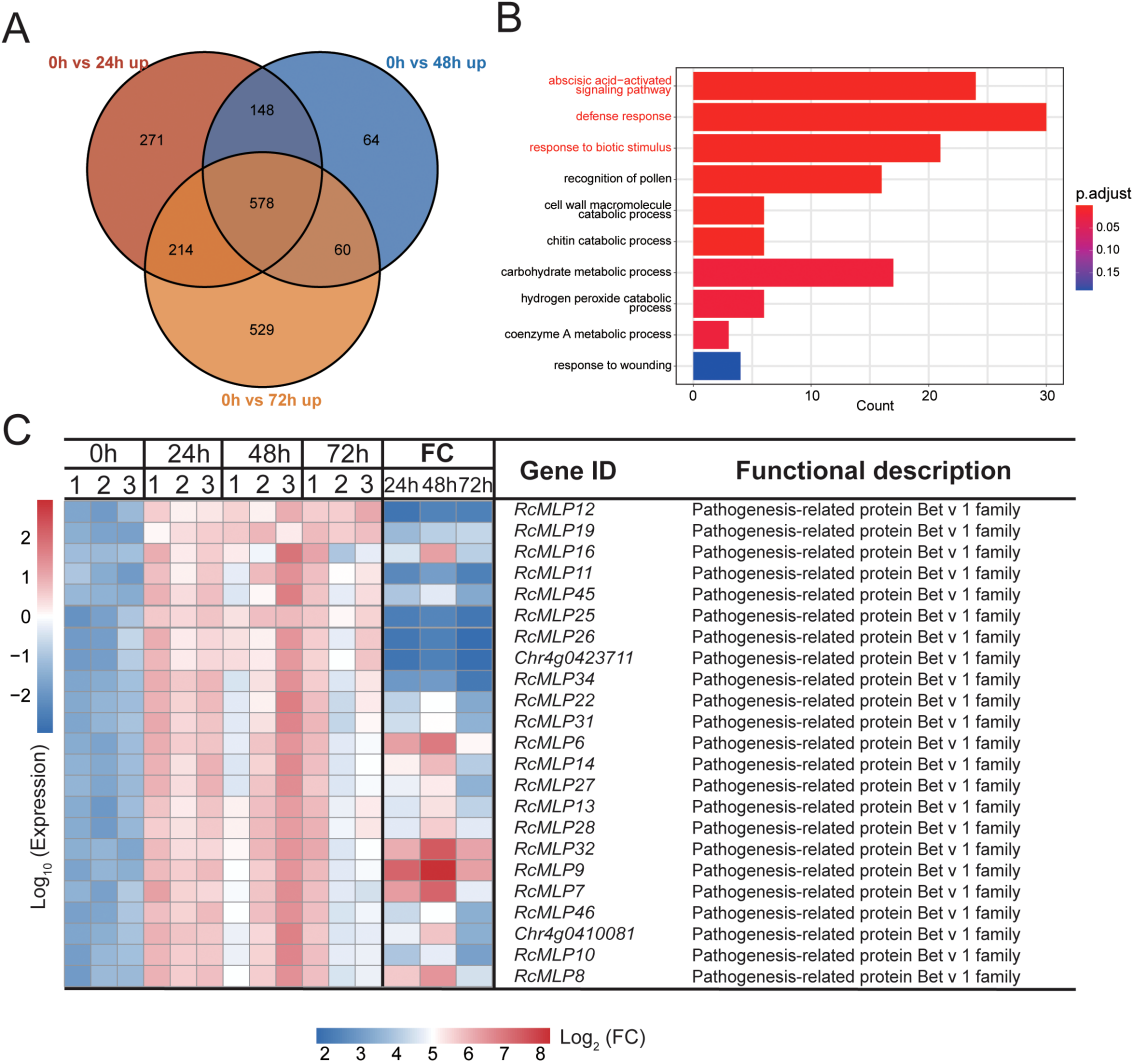
Identification of candidate *B. cinerea* infection-regulated rose genes. **(A)** Venn diagram depicting number and overlap among upregulated DEGs from each time point. **(B)** The bar plot illustrates the top 10 Biological Process (BP) GO terms enriched with commonly upregulated DEGs in **(A)**, highlighting the three most significant infection-related terms in red, and the genes enriched within these terms are considered potential candidates. **(C)** Heatmap depicting the expression patterns and protein domain annotations of the candidate genes identified in **(B)**. Data normalized using Z-score method. FC represents fold change between control and different infection time points.

By comparing the upregulated DEGs at 24 hpi, 48 hpi, and 72 hpi, 578 significantly upregulated DEGs were identified across all three time points (**Figure 2A**). GO enrichment analysis of these 578 DEGs revealed significant enrichment in three infection response-related pathways: the abscisic acid-activated signaling pathway, defense response, and response to biotic stimulus (**Figure 2B**). Protein domain analysis of the 31 DEGs annotated in three pathways (part of genes shared) revealed that the 23 genes with the pathogenesis-related protein Bet v 1 family (PF000407) domain, belonging to the *MLP* gene family (**Figure 2C**), suggesting their potential role in plant disease resistance. It is noteworthy that *RcMLP9*, *RcMLP7*, *RcMLP6*, *RcMLP32*, *RcMLP8*, and *RcMLP14* exhibited significant differential expression at 24 hpi with a log2 fold change >5 and FDR value < 0.01. Similarly, at 48 hpi, *RcMLP9*, *RcMLP32*, *RcMLP7*, *RcMLP6*, *RcMLP8*, *RcMLP16*, *RcMLP14*, *Chr4g0410081*, *RcMLP28*, *RcMLP13*, *RcMLP27*, and *RcMLP31* demonstrated significant differential expression and *RcMLP9*, *RcMLP32*, and *RcMLP6* were identified as significant differential expression genes at 72 hpi (**Supplementary Table S6**).

### 3.3 Systematic identification of *MLP* genes family in rose

In order to comprehensive study the roles of *MLPs* in resisting rose pathogens, we carried out the genome-wide identification of *MLP* gene family in rose. Utilizing the pathogenesis-related protein Bet v 1 family domain (PF000407, abbreviated as Bet v 1) as a signature motif, we identified 46 *RcMLP* genes in the rose genome (**Supplementary Table S7**). These genes are named sequentially from *RcMLP1* to *RcMLP46* based on their chromosomal positions. The members of the *RcMLP* gene family exhibit significant differences in amino acid length and physicochemical properties. The number of amino acids ranges from 124 (*RcMLP41*) to 227 (*RcMLP19*), molecular weight of proteins varies between 14118.77 (*RcMLP41*) and 25532.37 Da (*RcMLP19*), the theoretical isoelectric point ranges from 4.7 (*RcMLP38*) to 9.15 (*RcMLP39*), and the Grand Average of Hydrophobicity Index (GRAVY) values range from −0.611 (*RcMLP23*) to 0.113 (*RcMLP16*). Predictive analysis of subcellular localization indicates that all *RcMLPs* were located in the cytoplasm.

### 3.4 Phylogenetic analysis of the *RcMLP* gene family

**Figure 3:**
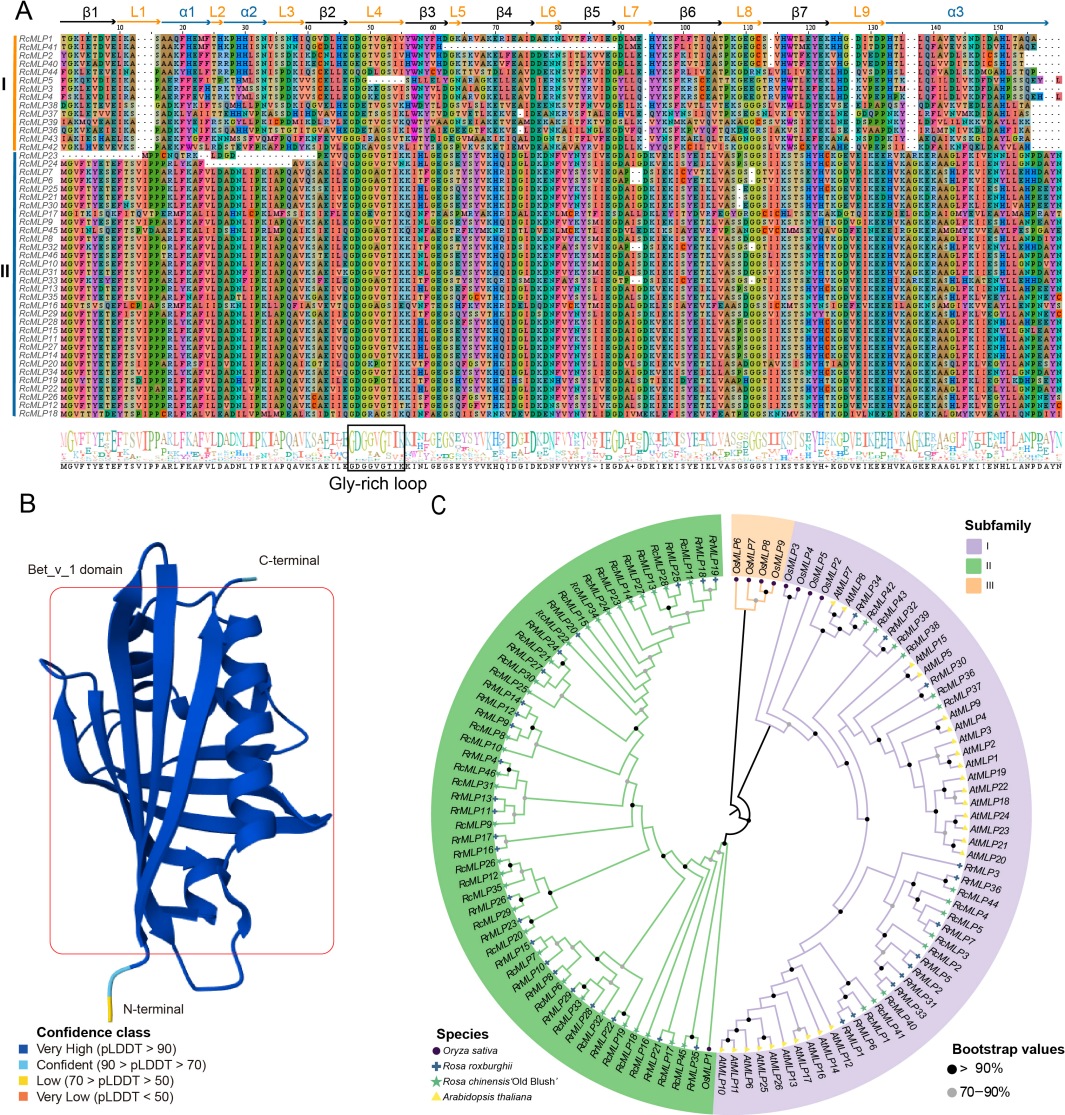
Evolution analysis of *RcMLPs* in rose. **(A)** The multiple sequence alignment of *RcMLPs* Bet v1 domain sequences. Sequence logo of Bet v1 domain, generated by WebLogo. We have marked the positions of the 3-D structure predicted in **(B)** atop the aligned 1D sequence diagrams. **(B)** The 3D structure model of Bet v1 domain predicted by AlphaFold3. **(C)** The ML phylogenetic tree of 117 *MLPs* from four species.

The conserved domains and phylogenetic relationships of RcMLP proteins were explored through multiple alignment Bet v1 domain sequences. The results of the multiple sequence alignments indicate that *RcMLP* could be distinctly divided into two groups, namely group I and group II (**Figure 3A**). The 3-D structural analysis of this domain reveals three α-helices (short α1 and α2, and a long, flexible α3), seven β-strands (β1 to β7), and nine loops (L1 to L9), as illustrated in **Figure 3B**, based on the AlphaFold database. Group I has some amino acid deletions compared to group II, including the absence of Valine (V), Isoleucine (I) and Proline (P) residues at positions 13-15 in the L1 loop, the absence of Aspartic acid (D) at position 94 in L7 loop, the absence of V at position 127 in L9 loop, and the absence of Alanine (A) at 136 and Glycine (G) at 137 in α3. Both group I and group II have a conserved Gly-rich loop GDG[G/T][V/A]G[T/S][I/V]K located in the L4 Loop, which connects β2 and β3, which is crucial for the endonuclease activity of MLP (Fernandes et al., 2013).

To study the evolutionary relationship between the *MLP* gene family in different plant species, we combined the protein sequences of 45 *RcMLPs* from rose, 36 *RrMLPs* from *R. roxburghii*, 26 *AtMLPs* from *A. thaliana* and 9 *OsMLPs* from rice (**Supplementary Table S12**) to construct a ML phylogenetic tree. The results showed that a total of 117 *MLP* genes can be phylogenetically divided into three subfamilies: I, II, and III (**Figure 3C**). 14 *RcMLP* genes in rose were clustered within group I, aligning with the members of group I depicted in **Figure 3A** and present across all four species, suggesting a possible common ancestry among them. 32 *RcMLP* genes were clustered within group II and this group is uniquely comprised of MLPs indigenous to the genus Rosa, with no homolog genes detected in *A. thaliana* or *O. sativa*. Group III is only found in rice and no any member were found for rose, indicating the evolutionary variations between monocotyledons and dicotyledons.

### 3.5 Gene duplication and collinearity analysis of *RcMLP* genes

**Figure 4:**
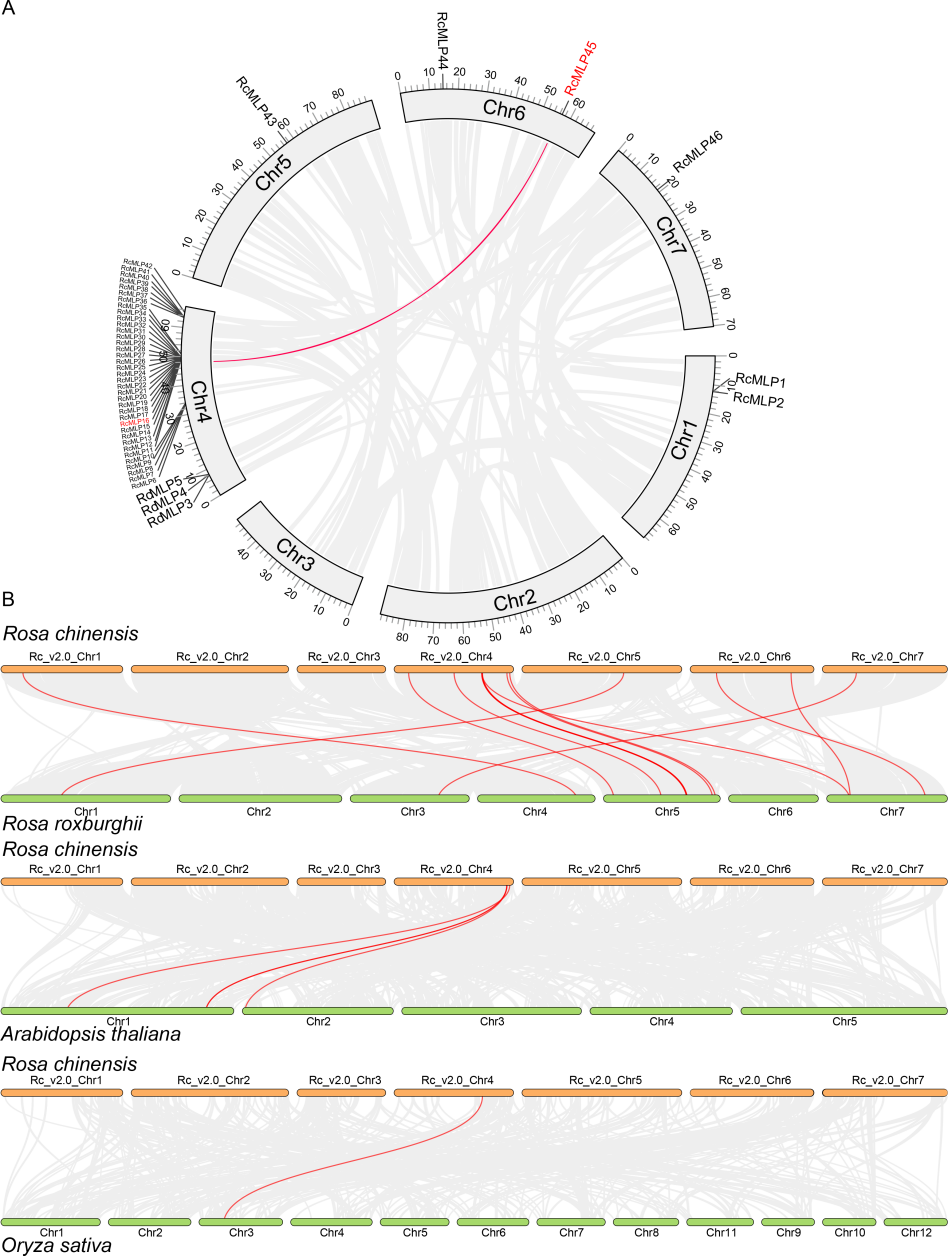
Collinearity analysis of *RcMLP* gene family. **(A)** Chromosomal locations and their synteny of *RcMLP* genes in rose. Gray lines indicate all synteny blocks in the rose genome and red line indicates segmental duplication of *RcMLP* genes. **(B)** Synteny analysis *RcMLP* genes between rose and *R. roxburghii*, *A. thaliana*, and *O. sativa*, respectively. The syntenic *MLP* gene pairs were highlighted in red lines.

Our genomic analysis of roses revealed that *RcMLP* genes are dispersed across five out of the seven chromosomes, with a significant clustering on chromosome 4 (**Figure 4A**). Using MCScanX, we discovered 33 tandem and 2 segmental duplicates in the rose *RcMLP* gene family (**Supplementary Table S8**), suggesting that tandem duplication events may have contributed to the expansion of the *RcMLP* gene family in rose genome. Of particular note in subfamily II (**Figure 3C**), tandem duplicated genes account for a substantial 84.38%, and an overwhelming 93.75% of these genes are clustered on chromosome 4, with the exception of *RcMLP45* and *RcMLP46*. This distribution strongly suggests that these RcMLPs have emerged from distinctive tandem duplication events which specific to genus Rosa. Furthermore, intraspecific collinearity analysis identified only one gene pair, *RcMLP16* and *RcMLP45*, suggesting a minor impact of segmental duplication on the expansion of *RcMLP* genes (**Figure 4A**). The Ka/Ks ratio, a measure of evolutionary pressure, was analyzed for *RcMLP* gene duplicates and results showed Ka/Ks ratios were below 1(**Supplementary Table S9**), suggesting purifying selection in *RcMLP* evolution.

To further explore the possible evolutionary processes of the *RcMLP* genes among species, we analyzed the collinearity of the *MLP* family genes between rose with *R. roxburghii*, *O. sativa*, and *A. thaliana*, respectively (**Figure 4B**). The collinearity analysis between *R. chinensis* and *R. roxburghii* indicated the *RcMLPs* has the most synteny with the *RrMLPs*, exhibiting a predominant one-to-one homozygosity. Comparisons with *A. thaliana* uncovered extensive of chromosomal rearrangements, identifying only four syntenic gene pairs: *RcMLP37*-*AtMLP17* (*AT1G70890*), *RcMLP36*-*AtMLP19* (*AT2G01530*), *RcMLP40*-*AtMLP16* (*AT1G70880*), and *RcMLP42*-*AtMLP8* (*AT1G24020*), and they were all in group I subfamily. Notably, there was only one collinear gene *RcMLP32*, classified under the group II subfamily, exhibited syntenic relationship with the rice gene *OsMLP1* (*LOC_Os03g18850*). These results demonstrated that the rapid evolution and contribution of species-specific genes tandem duplication in the expansion of the *MLP* gene family.

### 3.6 Gene structure, conserved motifs and promoter analysis *RcMLP* gene family

**Figure 5:**
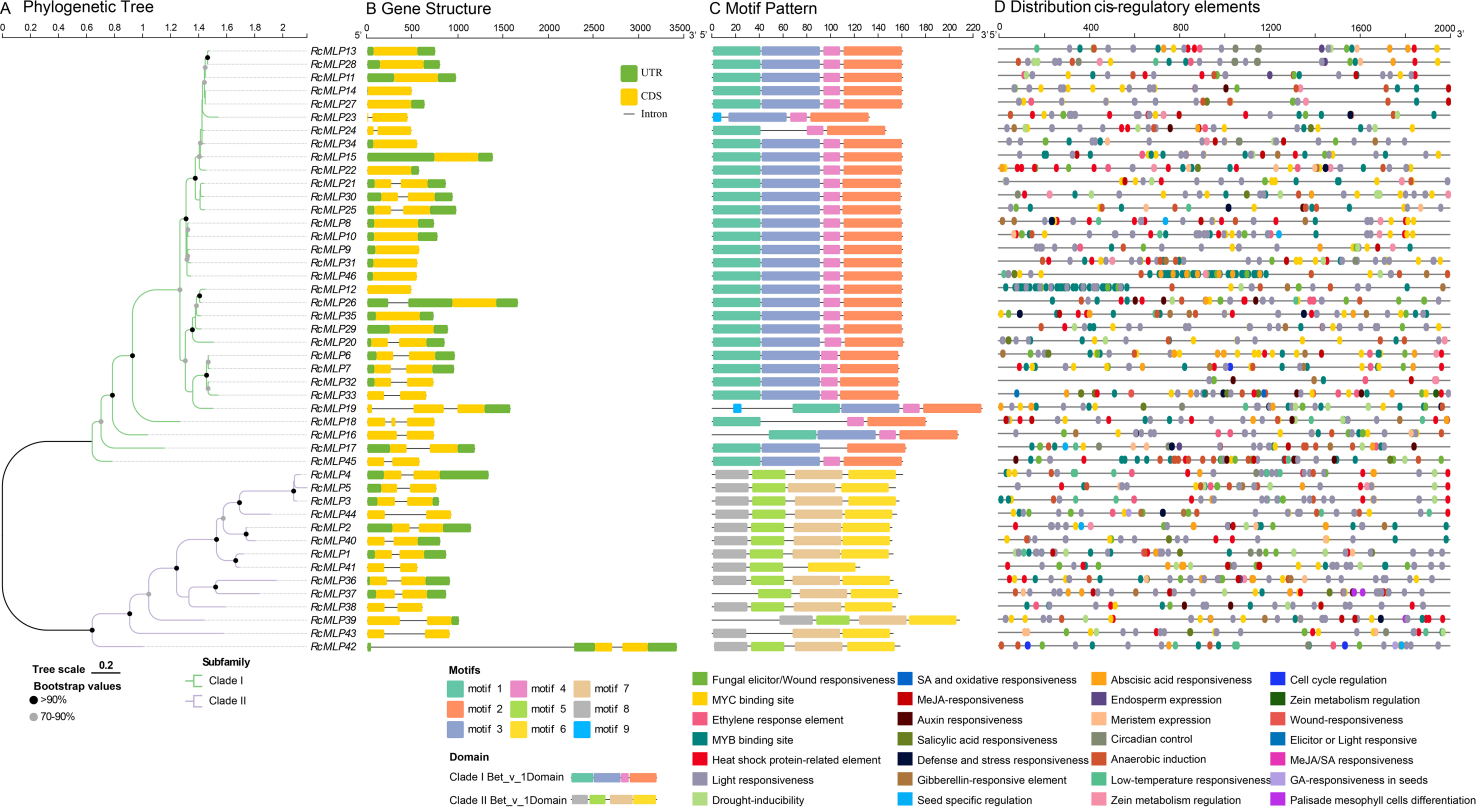
The phylogenetic tree, gene structures, protein motifs, and *cis*-regulatory elements analysis among *RcMLPs*. **(A)** The phylogenetic tree of *RcMLPs* was built with full-length protein sequences by the maximum likelihood (ML) method. **(B)** The gene structures of *RcMLP* gene family genes. Yellow boxes, green boxes and black horizontal lines displayed the CDS regions, UTR regions and introns, respectively. **(C)** The 9 conserved motifs of *RcMLP* gene family were represented by boxes with different colors. **(D)** *Cis*-regulatory elements (CREs) distribution of each *RcMLP* was shown.

We used the protein sequences of 45 *RcMLPs* from rose to construct a ML phylogenetic tree. The results showed that *RcMLP* genes can be phylogenetically divided into two subfamilies: I and II (**Figure 5A**; **Supplementary Figure S1**). To elucidate the structural attributes and potential functions of the *MLP* gene family in rose, we undertook a comprehensive analysis of gene structures and motif compositions. Exon/intron structure analysis revealed that among the 46 *RcMLPs*, the coding sequences (CDSs) of 30 (~65.22%) were interrupted by introns (**Figure 5B**). Of these, 27 *RcMLPs* has two exons, and 3 *RcMLPs* has three exons. These variations may have arisen from changes during gene duplication events. Genes clustered within the same subgroup exhibited similar exon/intron structures. Using motif-based sequence analysis tool (MEME), motifs were identified and sequence logos for motifs 1 through 9 were created to predict the structural features of the *RcMLP* proteins and to identify conserved amino acid residues (**Figure 5C**; **Supplementary Figure S2**). Notably, members of the same subgroup shared similar motif composition. The *RcMLP* proteins functional domain Bet_v_1 were also shown in **Supplementary Figure S3**. The most of *RcMLPs* in subfamily I has motifs 1 to 4, with only two genes *RcMLP19* and *RcMLP23* has motif 5, whereas the majority of *RcMLPs* in the subfamily II has motifs 6 to 9. This result was consistent with previous studies (Feki et al., 2023).

Furthermore, we predicted *cis*-regulatory elements (CREs) within the 2-kilobase pair upstream regions of the *RcMLP* genes using PlantCARE. A total of 1,495 CREs were predicted in the promoter regions of *RcMLPs* (**Supplementary Table S10**) and the representative CREs were shown in **Figure 5D**. All CREs can be divided into four main categories. The first category is related to stress responsiveness (627), mainly including MYC binding site (132, 21.05%), MYB binding site (122, 19.46%), stress response element (102, 16.27%), anaerobic induction (92, 14.67%), fungal elicitor/wound responsiveness (52, 8.29%), and drought inducibility (41, 6.54%). Our focus is on the fungal elicitor/wound responsive elements (W Box), which are associated with 32 *RcMLP* genes. Notably, the genes *RcMLP45*, *RcMLP46*, and *RcMLP7* contain at least three of these elements each. The second category is related to hormone responsiveness (323), including abscisic acid (ABA) responsiveness (123, 38.08%), MeJA responsiveness (67, 20.74%), salicylic acid (SA) responsiveness (53, 16.41%), gibberellin (GA) responsiveness (34, 10.53%), and ethylene response element (28, 8.67%). Among them, the promoter regions of 41 and 25 *RcMLPs* abundantly displayed ABA responsiveness (ABRE) and MeJA responsiveness (CGTCA motif and TGACG motif), respectively. The *RcMLP12* gene contained the highest number of ABA regulatory elements with totaling of 11 elements, while the *RcMLP37* gene has the most MeJA regulatory elements, with 8 elements. Additionally, we found that the *RcMLP17* and *RcMLP24* genes contained the greatest variety of hormone responsiveness elements, amounting to six types, a broad spectrum of functions within roses. The third category is related to light responsiveness (459). CREs associated with light-responsive elements (such as G-box Box 4, GT1-motif, TCT-motif et al.) were present in the promoter region of all *RcMLPs*. The remaining category is related to growth and development responsiveness (86), mainly including zein metabolism regulation (25), meristem (22), and circadian (21), and the genes *RcMLP28*, *RcMLP13*, and *RcMLP17* contain more than five of these elements. In summary, these analyses collectively offer insights into the physiological functions of *RcMLPs* in rose under both normal and stress conditions.

### 3.7 Regulatory network analysis of *RcMLP* genes

**Figure 6:**
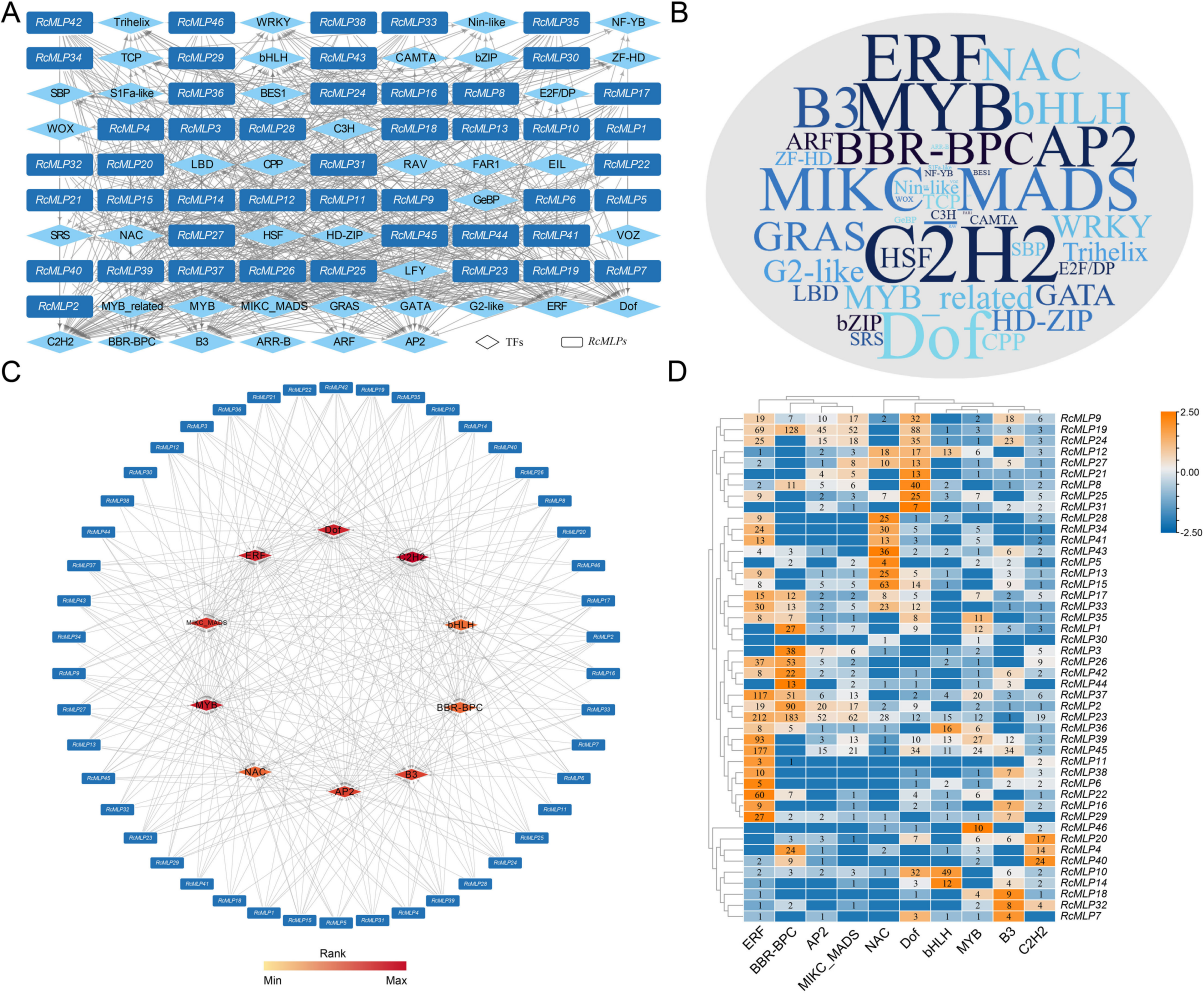
The putative TFs regulatory network analysis of *RcMLPs*. **(A)** All putative transcription factors (TFs) were represented by diamond nodes, *RcMLPs* were represented by rectangle nodes. The putative TFs and related mediated *RcMLPs* were linked by grey lines. **(B)** Wordcloud for TFs. Font size is positively correlated with the gene number of corresponding TFs. **(C)** Top 10 highly enriched and targeted *RcMLPs* were shown and the darker the color shows highly enriched. **(D)** The number of top 10 enriched TFs distributions across the targeted *RcMLP* genes.

To predict the transcription factors (TFs) potentially regulating the *RcMLP* genes, we conducted an analysis of the CREs within the promoter regions of these genes using the Plant Transcriptional Regulatory Map. Our analysis identified a total of 41 putative TFs with 4,360 binding sites, suggesting a complex regulatory landscape for the *RcMLP* gene family (**Figure 6A**; **Supplementary Table S11**). The TFs were abundant in C2H2 (42), MYB (37), Dof (36), ERF (36), MIKC_MADS (33), AP2 (31), and B3 (31) (**Figure 6B**), and the least abundant TF families distributed only a few members, such as EIL (2), FAR1 (2), RAV (2), VOZ (2), and LFY (1) (**Supplementary Table S11**). According to the prediction results, *RcMLP39* has the highest number of regulatory factors among all *RcMLP* genes, with a total of 24 TFs, followed by *RcMLP19* and *RcMLP37*, each having 23 TFs (**Supplementary Table S11**). In addition, the top ten enriched TF gene families predicted to be involved in regulating *RcMLPs* were identified, including C2H2, MYB, Dof, ERF, MIKC_MADS, AP2, B3, BBR-BPC, NAC, and bHLH (**Figure 6C**). Notably, the ERF family showed the broadest regulatory influence across various *RcMLP* members with the most enriched TFs (total 1,040 members) (**Figure 6D**).Our predictions aligned with the known RhERF005/RhCCCH12–RhPR10.1 module, which is implicated in cytokinin-induced defense responses to *B. cinerea* in roses (Liu et al., 2024).Overall, the predicted TFs regulatory network of *RcMLPs* suggests their potential roles in responses to biotic stresses and network interactions.

### 3.8 Expression patterns of *RcMLP* gene family response to *B. cinerea*se

**Figure 7:**
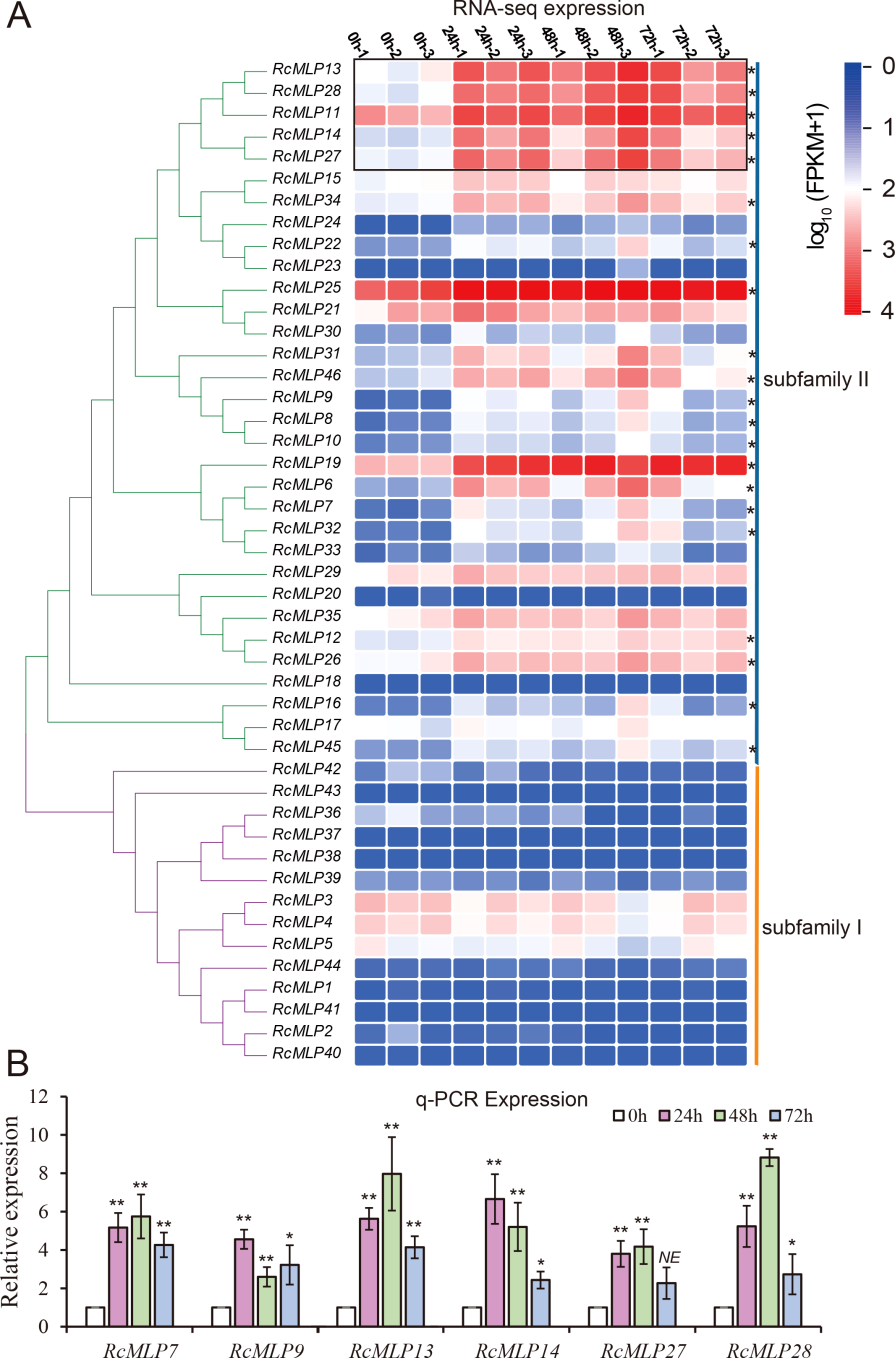
Expression patterns of *RcMLP* family at different time points during *B. cinerea* infection of rose. **(A)** The heatmap shows the expression levels of 46 genes encoding MLP gene family in rose petals at 0 hours (uninfected control) and at 24, 48, and 72 hours post-*B. cinerea* infection. **(B)** qRT-PCR results of eight selected *RcMLPs* genes under *B. cinerea* infection stress. * represents p < 0.05, ** represents p < 0.01, *** represents p < 0.001, **** represents p < 0.0001.

*MLPs* enhance plant resistance to pathogens by inducing the expression of disease resistance-related genes, playing a crucial role in plant responses to diseases. At all treatment time, 21 *RcMLP* genes were significantly upregulated, and *RcMLP9* has the highest fold change at 48 hpi (**Figure 2C**). Phylogenetic analysis of the 46 *RcMLPs* revealed that in subfamily II, 19 genes were significantly upregulated at all three-time points treatment (**Figure 7A**), accounting for 90.5% of the significantly upregulated genes, suggesting that this clade may play a key role in responding to *B. cinerea* infection. Among them, *RcMLP13*, *RcMLP28*, *RcMLP11*, *RcMLP14*, and *RcMLP27* exhibited relatively high expression levels at all treatment time and were clustered together. Notably, *RcMLP13*, *RcMLP14*, *RcMLP27*, and *RcMLP28* at 48 hpi and *RcMLP14* at 24 hpi had a fold change more than 10. To verify the expression data obtained by RNA sequencing, qRT-PCR was used to examine the expression patterns of six *RcMLP* genes selected under *B. cinerea* infection stress (**Figure 7B**). The expression levels of the *RcMLP* genes under *B. cinerea* infection stress were largely consistent with the transcriptome data.

## 4 Discussion

Despite the significant economic implications of gray mold disease on roses, the intricacies of rose defense mechanisms during *B. cinerea* infection are not well understood. In this study, we conducted transcriptome sequencing on petals of the resistant rose cultivar at 24, 48, and 72 hpi with *B. cinerea* to elucidate the temporal defense responses (**Figure 1**). The analysis revealed that 578 DEGs were significantly upregulated at all three time points (**Figure 2A**). Notably, these DEGs were enriched in the *MLP* gene family containing the Bet_v_1 domain (**Figure 2B**).

The *MLP* gene family is crucial in mediating both biotic and abiotic stress responses in plants (Agarwal and Agarwal, 2014; Liu and Ekramoddoullah, 2006). The Bet_v_1 domain, a distinct feature of MLP proteins, is integral to their function (Kofler et al., 2014; Song et al., 2020). This domain facilitates small-molecule binding, thereby regulating the immune system and enabling *MLP* to participate in defense mechanisms against various biotic stresses (Jain et al., 2016; Radauer et al., 2008). *MLP* were initially discovered in the *Papaver somniferum* and have since been identified in multiple plant species (Song et al., 2020). For instance, 10 *PR-10* genes have been identified in *P. bretschneideri* (Su et al., 2024a), and 13 members have been identified in *Phaseolus vulgaris* (Feki et al., 2023). These gene families play critical roles in plant growth, development, and responses to both biotic and abiotic stresses. The *MLP* genes likely enhance plant disease resistance by regulating specific defense pathways, such as allergen signaling and PR protein responses (Hendrich et al., 2024; Melnikova et al., 2024; Metwally et al., 2024). Furthermore, some *MLP* genes may work synergistically with other defense-related genes, forming a complex immune network. These genes not only function in *B. cinerea* resistance but may also confer broad-spectrum resistance to other pathogens (Castro et al., 2016; Varveri et al., 2024). Similarly, research has shown that *MLP* genes play pivotal roles in various stresses by modulating or participating in plant defense pathways to enhance resistance (Longsaward and Viboonjun, 2024; Sun et al., 2024, Sun et al., 2023). In *Nicotiana benthamiana*, *NbMLP28* is upregulated through the jasmonic acid (JA) signaling pathway, contributing to plant defense responses and significantly enhancing resistance to Potato virus Y, improving disease resistance in tobacco (Song et al., 2020). In peach, *PpMLP10* is significantly upregulated during cold storage in response to cold stress, and its overexpression enhances membrane stability and reduces damage, increasing cold tolerance in peach (Ma et al., 2024). In *Brassica napus*, *BnMLP6* is a key defense gene, encoding a protein that interacts with NPF5.12 at the plasma membrane and endoplasmic reticulum, fortifying the plant’s root barrier by increasing suberin deposition to limit *Verticillium longisporum* infection and spread (Dolfors et al., 2024). These findings indicate that the *MLP* gene family broadly participates in plant responses to diverse biotic and abiotic stresses.

However, research on *MLP* in roses remains limited. To further investigate the role of *MLP* genes in rose’s response to *B. cinerea* infection, we conducted a systematic identification of the *MLP* gene family in roses and identified 46 *MLP* genes (**Figure 3**). Multiple sequence alignment revealed that *MLP* genes across different evolutionary branches retain the core Bet_v_1 domain, indicating that *MLP* genes have conserved biological functions across evolution, participating in the regulation of plant immune responses to pathogens (Schenk et al., 2009). Phylogenetic analysis of the Rosa genus has revealed a unique subset of *MLP* genes, Subfamily II, which are indigenous to Rosa and not found in *A. thaliana* or *O. sativa* (**Figure 3**). This suggests a distinct evolutionary path for MLP genes in Rosa. The high proportion of tandem duplicated genes within Subfamily II, particularly their clustering on chromosome 4 (**Figure 4A**), indicates that these genes likely emerged from specific tandem duplication events unique to Rosa. This clustering and duplication could be linked to the genus’s adaptation, possibly contributing to antifungal capabilities. Additionally, functional characterization through gene expression, indicating the group II subfamily genes tends to respond to *B. cinerea* infections, suggesting their roles in the adaptation and survival of Rosa species in their natural habitats. The absence of these genes in *A. thaliana* and *O. sativa* implies that their functions may be specific to Rosa or have evolved for unique roles within this genus. Further research, including a broader species comparison and functional studies, is needed to substantiate this hypothesis and understand the role of these genes in the biology of Rosa species. Comparative genomics across more Rosaceae family members and other plant families could provide insights into the conservation and divergence of these genes.

Additionally, *cis*-regulatory element analysis revealed that these genes contain functional elements related to hormone response, environmental response, and growth and development (**Figure 5**). Particularly, the presence of ABA response elements (ABRE) suggests that these genes could be targets of ABA signaling. Previous studies have demonstrated that *MLP* genes are induced under abiotic stress conditions and enhance plant resistance by promoting ABA signaling (Wang et al., 2016). *PsnMLP5* was activated after ABA treatment, suggesting its regulatory role under abiotic stress conditions via the ABA pathway (Sun et al., 2024). In tobacco, *NtMLP423* regulates drought resistance via the ABA pathway, and its overexpression significantly improves drought tolerance (Liu et al., 2020). To further analysis how the *MLP* gene family responds to *B. cinerea* infection, we predicted the transcription factor binding sites in *MLP* genes and found that NAC, bHLH, BBR-BPC, and ERF gene families might be involved in regulating *MLP* genes (**Figure 6**). These transcription factors are likely to work in concert with the *MLP* gene family to regulate plant immune responses and play key roles in specific signaling pathways, providing new insights into the regulatory mechanisms of rose’s response to *B. cinerea* infection (Liu et al., 2020; Song et al., 2020). The ERF family of transcription factors (TFs) exhibited the broadest regulatory influence across various *RcMLP* members, which aligns with previous studies that have implicated ERF TFs in the regulation of plant defense responses (Deng et al., 2024; Liu et al., 2024).

In the expression analysis results, the upregulation of *RcMLP* genes in response to *B. cinerea* highlights their critical role in rose defense mechanisms. The significant expression of subfamily II genes, particularly *RcMLP13*, *RcMLP14*, *RcMLP27*, and *RcMLP28* (**Figure 7**), indicates their potential as key regulators in the resistance against gray mold disease. Future studies should focus on functional validation of these genes and their roles in other environmental stresses, offering new strategies and directions for crop improvement and breeding projects.

## 5 Conclusions

This study investigates the transcriptomic profiling of the resistant rose cultivars, ‘Yellow Leisure Liness’, in response to *Botrytis cinerea* infection, revealing the pivotal role of the *RcMLP* gene family in fungal resistance. We systematically identified and classified 46 *RcMLP* genes into two subfamilies, and gene duplication analysis indicates that tandem duplication contributes to the expansion of the *MLP* gene family, especially for group II subfamily. All *RcMLP* genes were showed evidence of purifying selection. Detailed structural and promoter analyses, along with the regulatory network construction, reveals a complex interplay of transcription factors, particularly the ERF family, in modulating *MLP* gene. Expression analysis confirms that the upregulation of 21 *RcMLP* genes in response to *B. cinerea* are all belonging to the group II subfamily, suggesting their potential roles in disease resistance. This comprehensive analysis of the rose transcriptome and *MLP* gene family enhances our understanding of the molecular mechanisms underlying rose resistance to gray mold disease and provides a foundation for future breeding efforts.

## Data Availability

The datasets presented in this study can be found in online repositories. The names of the repository/repositories and accession number(s) can be found in the article/**Supplementary Material.**

## Supplementary material

Supplementary Figure 1 An maximum-likelihood tree showing the MLP phylogenetic topology.

Supplementary Figure 2 Motif sequence LOGO found in RcMLP proteins.

Supplementary Figure 3 RcMLP proteins domains.
