# Supplementary figures and images for: Identification and analysis of major latex protein (MLP) family genes in Rosa chinensis responsive to Botrytis cinerea infection by RNA-seq approaches
Source: Front Plant Sci. 2024 Dec 13;15:1511597. doi: 10.3389/fpls.2024.1511597 (PMC11671256; doi:10.3389/fpls.2024.1511597)

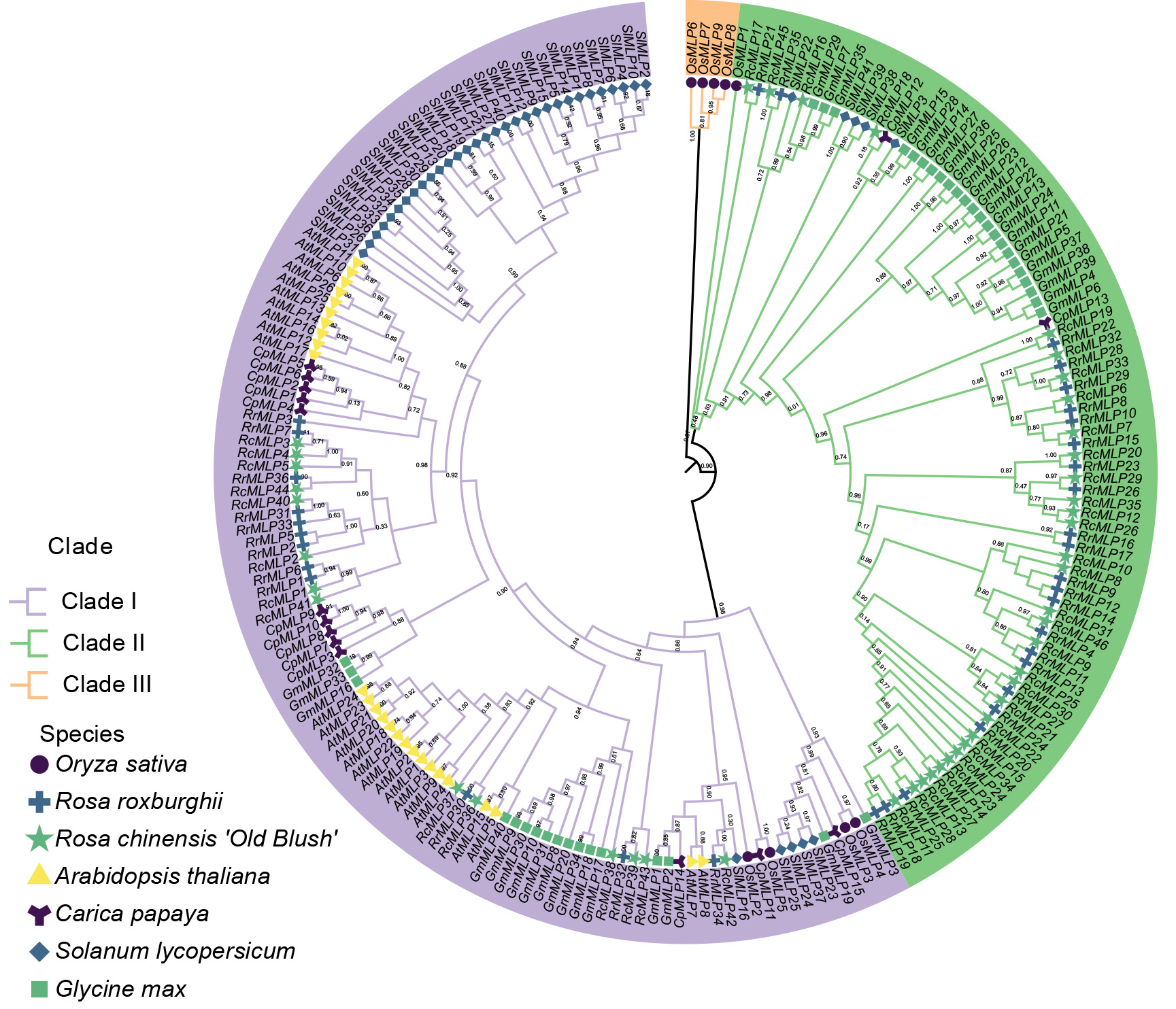

Supplement: Supplementary Figure 1 — An maximum-likelihood tree showing the MLP phylogenetic topology. [file Image1.jpeg]

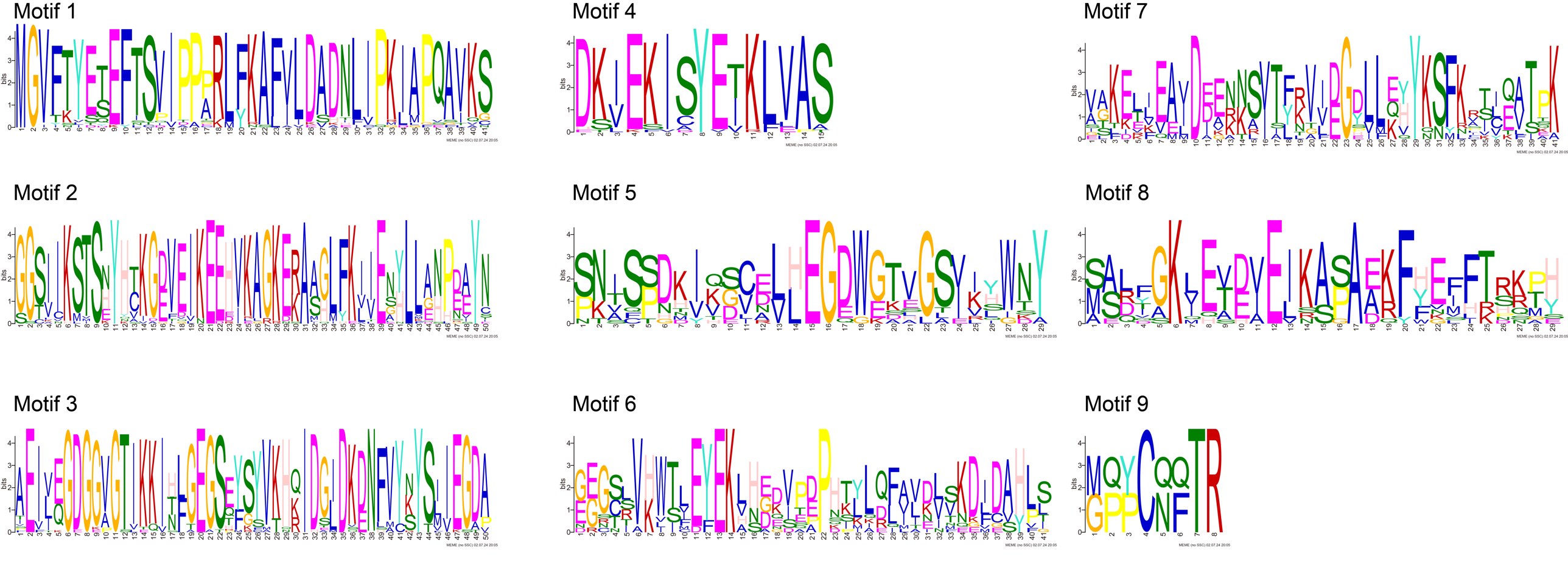

Supplement: Supplementary Figure 2 — Motif sequence LOGO found in RcMLP proteins. [file Image2.jpeg]

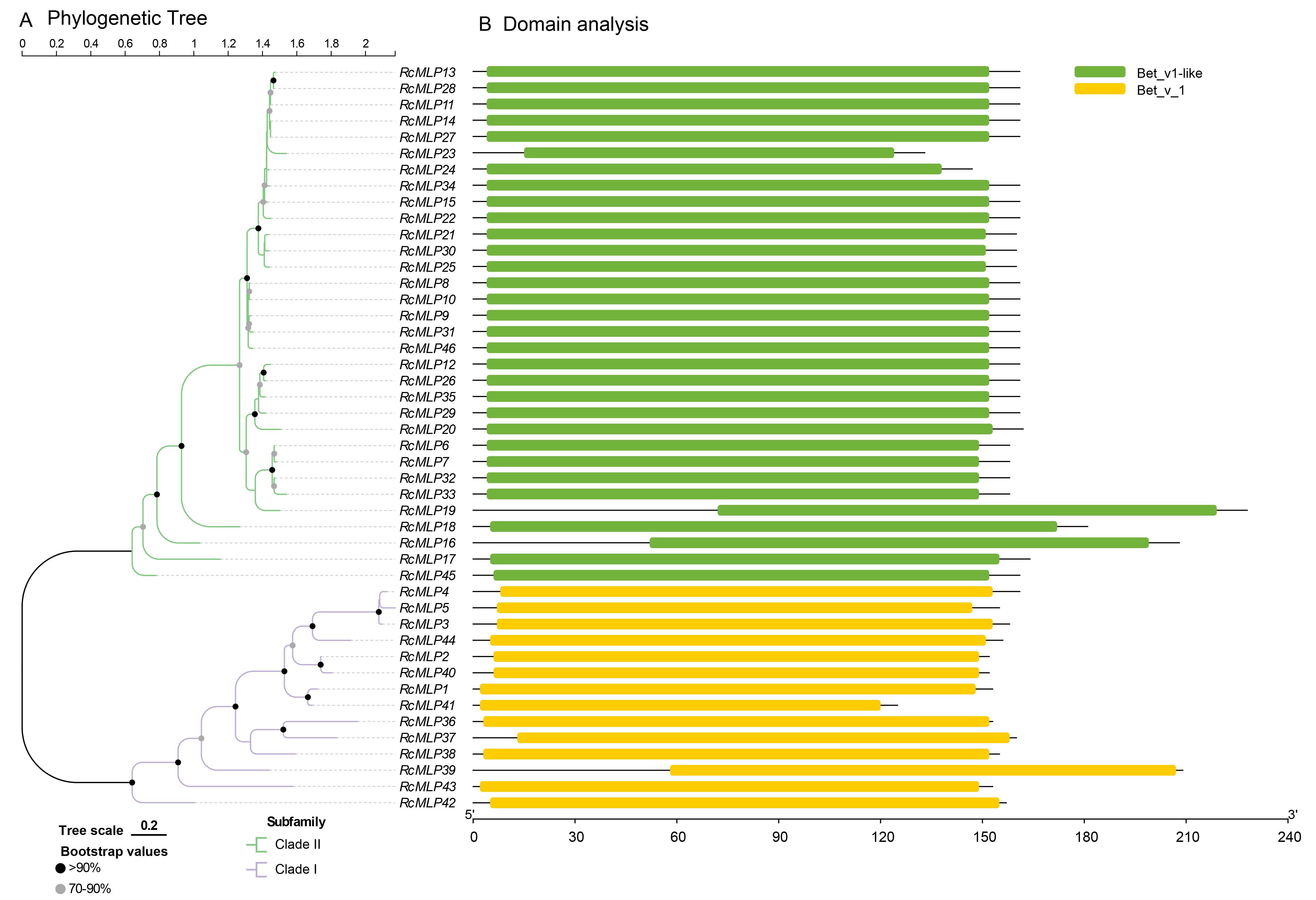

Supplement: Supplementary Figure 3 — RcMLP proteins domains. [file Image3.jpeg]
